# A general co-expression network-based approach to gene expression analysis: comparison and applications

**DOI:** 10.1186/1752-0509-4-8

**Published:** 2010-02-02

**Authors:** Jianhua Ruan, Angela K Dean, Weixiong Zhang

**Affiliations:** 1 Department of Computer Science, The University of Texas at San Antonio, One UTSA Circle, San Antonio, TX 78249, USA; 2 Department of Computer Science and Engineering, Washington University in St Louis, One Brookings Dr, St Louis, MO 63130, USA; 3 Department of Genetics, Washington University School of Medicine, Campus Box 8232, St Louis, MO 63110, USA

## Abstract

**Background:**

Co-expression network-based approaches have become popular in analyzing microarray data, such as for detecting functional gene modules. However, co-expression networks are often constructed by ad hoc methods, and network-based analyses have not been shown to outperform the conventional cluster analyses, partially due to the lack of an unbiased evaluation metric.

**Results:**

Here, we develop a general co-expression network-based approach for analyzing both genes and samples in microarray data. Our approach consists of a simple but robust rank-based network construction method, a parameter-free module discovery algorithm and a novel reference network-based metric for module evaluation. We report some interesting topological properties of rank-based co-expression networks that are very different from that of value-based networks in the literature. Using a large set of synthetic and real microarray data, we demonstrate the superior performance of our approach over several popular existing algorithms. Applications of our approach to yeast, Arabidopsis and human cancer microarray data reveal many interesting modules, including a fatal subtype of lymphoma and a gene module regulating yeast telomere integrity, which were missed by the existing methods.

**Conclusions:**

We demonstrated that our novel approach is very effective in discovering the modular structures in microarray data, both for genes and for samples. As the method is essentially parameter-free, it may be applied to large data sets where the number of clusters is difficult to estimate. The method is also very general and can be applied to other types of data. A MATLAB implementation of our algorithm can be downloaded from http://cs.utsa.edu/~jruan/Software.html.

## Background

The vast amount of available high-throughput gene expression data has provided excellent opportunities for studying gene functions on a global scale. Since genes on the same pathways or in the same functional complex often exhibit similar expression patterns under diverse temporal and physiological conditions, one common practice in microarray data analysis is to cluster genes according to their expression similarities. The clusters can then be analyzed in several ways, such as promoter analysis or gene ontology analysis [1]. Since clustering procedures are often subjective, and usually ignore the detailed relationships among genes, the biological insight obtained from clustering results is often limited. Alternatively, many studies have attempted to construct gene regulatory networks from microarray data using methods such as linear models [2], Bayesian networks [3] and Boolean networks [4]. These approaches, however, are successful only on rare cases where the system is well constrained and the training data is sufficiently large.

Recently, as a tradeoff between the crude cluster analysis and detailed network modeling, gene co-expression networks have become a rapidly developing area of study and many interesting results have been obtained [5-20]. A gene co-expression network is an undirected graph, where the graph nodes correspond to genes, and edges between genes represent significant co-expression relationships [10,18]. Compared to regulatory networks, a gene co-expression network does not attempt to distinguish direct gene interactions from indirect ones; on the other hand, a gene co-expression network contains gene neighborhood relations that are usually overlooked in cluster analysis [21]. gives an interesting geometric interpretation of gene co-expression networks and connects co-expression network analysis to traditional microarray data analysis techniques. Databases of gene co-expression networks for several model organisms have been constructed from large numbers of microarray data sets [22,23].

One of the most important applications of gene co-expression networks is to identify functional gene modules, which often manifest themselves as dense subnetworks. While earlier studies attempted to apply clustering algorithms directly to the adjacency matrices of networks in order to partition network nodes into groups [10,14], later studies have relied on graph partitioning algorithms or special purpose algorithms for identifying subnetworks of certain properties [5-7,11,17,18,20]. These studies have provided many interesting biological results; however, none of them has demonstrated that the network-based methods for detecting functional modules can significantly outperform the conventional cluster analysis, partially due to the lack of an unbiased metric for quantitatively evaluating the functional significance of gene modules. In principle, nearly all the previous gene co-expression network construction methods fall into two categories: those that utilize the similarity values (value-based) [5-16,24], and those that utilize the rank-transformed similarities (rank-based) [17,18,20]. In the value-based (rank-based) method, two genes are connected if the (rank-transformed) similarity between their expression profiles is above a certain threshold, which can be chosen in an ad hoc manner or semi-automatically. Similarities may be measured by Pearson correlation coefficient or other metrics. Some researchers proposed to use conditional correlations to partially remove indirect links [24]. Thresholds are usually chosen using an ad hoc methods [16], or by controlling statistical significance of similarities [10,19]. A semi-automatic method has also been proposed based the topological difference between real and random co-expression networks [8]. In addition, several methods have been proposed to adjust raw similarity values based on neighborhood information such as shortest path connections or topological overlaps, which usually result in weighted gene co-expression networks [6,16]. Most of the existing studies used the value-based method, while the relative advantages and disadvantages of the rank-based or the value-based network construction methods have not been rigorously examined.

A number of studies have analyzed the topological properties of gene co-expression networks [10-13,18,25], and have shown that gene co-expression networks have the well-known small-world and scale-free properties, similar to many other biological networks and real-world networks [26,27]. On the other hand, these studies have also reported that gene co-expression networks differ from other types of biological networks in several important aspects, such as the characteristic node degree and hierarchical organization. However, most of the conclusions were drawn from value-based co-expression networks. In fact, we will show that the rank-based network has all the common topological properties of the other biological networks, and is significantly different from the value-based network.

In this work, we propose a general co-expression network-based approach for analyzing microarray data, for both genes and samples. Our approach consists of a method for rank-based network construction, a parameter-free graph partitioning algorithm for module discovery and a novel reference network-based metric for module evaluation. We compare our approach with the existing methods, and show several real applications of our approach.

We first introduce a simple rank-based method to construct co-expression networks, and compare the topologies of value-based and rank-based gene co-expression networks. We find that the rank-based network significantly differs from the value-based network in several important aspects, and we argue that the former is able to better capture the global topology of the underlying biological system, including both strongly and weakly co-expressed modules, while the latter is dominated by the most strongly co-expressed modules.

Second, we introduce an efficient graph partitioning algorithm, *Qcut*, to identify relatively dense modules, also known as communities, in the rank-based networks. Compared to other graph partitioning algorithms, *Qcut *is parameter free, as it uses an objective function called modularity to automatically determine the optimal partitioning and the number of partitions [28]. We have recently shown that the method can be used to discover natural communities from social networks and to predict protein complexes from protein-protein interaction networks [28], while its effectiveness in finding intrinsic modular structures from gene expression data has not been thoroughly studied.

Third, we propose a novel metric for comparing different module detection algorithms in terms of the overall functional significance of the gene modules being identified. We introduce the concept of a reference network, which can be obtained from other information sources, such as protein-protein interaction networks or ChIP-chip data. This metric is then used to quantify the performance of a gene module detection algorithm by the agreement between the modules identified from the co-expression network and the modular structure of the reference network. It is important to note this metric is not biased by the number of modules and the module size distribution.

We assessed our approach for finding gene modules on a large set of synthetic microarray data with known modular structures as well as several real data sets. On synthetic data, our method correctly predicted the number of modules, and performed significantly better than several popular clustering algorithms and one of the best graph partitioning algorithms, the Markov Clustering algorithm [29]. On real data, we evaluated the performance of our method using both a popular (but in our opinion biased) metric based on gene ontology enrichment scores, and the aforementioned reference network-based metric. We show that our method can significantly outperform the existing algorithms according to all the metrics used.

We also applied our method to construct and analyze a sample co-expression network using microarray data of normal and cancerous T and B cells including diffuse large B-cell lymphoma (DLBCL) [30]. Remarkably, our method almost perfectly separated the different cell types into their own subnetworks without any prior knowledge. We also re-discovered the two known subtypes of DLBCL, where one subtype has a much lower survival rate than the other.

Finally, we report several interesting results that may worth further investigation. In yeast, we identified many gene modules that are both strongly co-expressed and co-regulated; among them, a small module where majority genes have no known functions may be involved in telomere maintenance. In the sample co-expression network, we discovered a tumor cell module that seems to be a new subtype of DLBCL that is associated with the lowest survival rate among all DLBCL patients.

## Results and Discussion

### Co-expression network construction and topological analysis

#### Constructing co-expression networks

**Figure 1:**
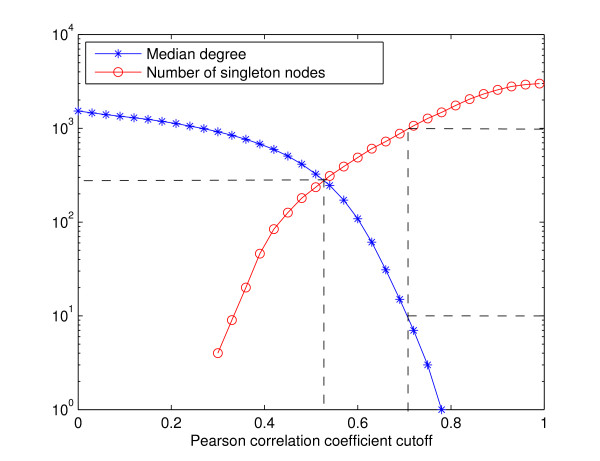
**Median degree and number of singleton nodes in a value-based yeast co-expression network**. Horizontal axis: the Pearson correlation coefficient threshold for the value-based network construction. Left vertical axis: median number of co-expression links per gene. Right vertical axis: number of genes without a co-expression link.

Most of the existing co-expression analyses construct value-based networks. We believe that the value-based methods are significantly limited by their use of a homogeneous threshold for all the genes in the network. In reality, genes in different functional pathways may be regulated by different mechanisms, and therefore may exhibit different patterns of co-expression. In particular, genes in one functional pathway may be strongly mutually co-expressed, while genes in another functional pathway may be only weakly co-expressed. As a result, if we choose a stringent global threshold, many genes in the weakly co-expressed pathway may be disconnected. On the other hand, if we attempt to connect the weakly co-expressed genes into the network, the threshold may become so low that the genes in the strongly co-expressed pathway may have many links to genes in other pathways, making further analysis difficult. For example, as shown in Figure 1, to construct a co-expression network for the 3000 yeast genes that we will see in the next subsection, if we allow only 10% of the genes to have no connections, most genes will have more than 300 connections, while if we reduce the median degree to 10, more than one third of the genes will have no connection at all.

To deal with this problem, we propose a simple rank-based method to construct co-expression networks. We first calculate the Pearson correlation coefficient (or some other similarity measure) between every pair of genes. For each gene *g_i_*, we rank all other genes by their similarity to *g_i_*. We then connect every gene to the *d *genes that are most similar to it. Compared to the value-based method, the rank-based method essentially uses different local similarity threshold for different genes. It is important to mention that even with a fixed *d*, the number of connections for different genes is not constant. This is because of the asymmetric nature of the ranking. In other words, the rank of gene *i *with respect to gene *j *is not necessarily equal to the rank of gene *j *with respect to gene *i*. Therefore, although gene *i *has only *d *genes on its top-*d *list, other genes that are not on *i*'s list may list *i *as one of their top-*d *genes. The mean degree is between *d *and 2*d*, the minimum degree is *d*, and the maximum degree can be as large as *n *- 1, with *n *being the number of genes in the network.

**Figure 2:**
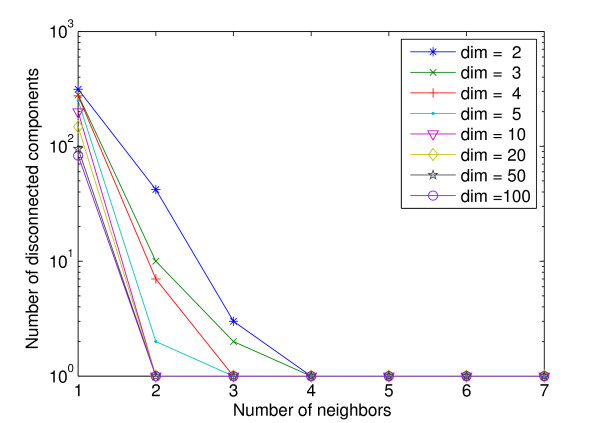
**Connectivity of rank-based co-expression networks on random data**. Each data set contains 1000 random geometric points in a certain number of dimensions, generated using the standard Gaussian distribution. Y-axis shows the number of disconnected components in the co-expression network constructed by the rank-based approach.

The rank-based method may appear to be limited by a similar drawback of the value-based method - the former uses a global rank threshold and the latter uses a global value threshold for all genes. However, as we discussed above, in the rank-based network, different genes can have different number of connections, even though all genes have the same rank threshold, because of the asymmetric nature of ranking. More importantly, our objective is not to identify *all *co-expressed genes for each gene, but to construct a sparse network such that the modular structure of the system can be successfully identified. To achieve this, a good co-expression network needs to have the following two properties: (i) there are very few false-positive connections, and (ii) nodes within modules are well connected into a single component, while connections between modules are sparse. A value-based network can hardly provide the two properties simultaneously, for reasons given above. In contrast, the key idea in our rank-based method is that by using a uniform small value of *d*, we ensure that (i) the network only contains highly reliable edges, and (ii) each module of the network is (almost) fully connected into a single component, both theoretically and empirically (see below). As in most clustering algorithms, we assume that gene expressions in different modules are generated by different distributions, while gene expressions in the same module are generated by a common (unknown) distribution. Therefore, the rank-based sub-network of genes from the same module is a nearest neighbor graph constructed on a set of random geometric points. Theoretically, it is known that a nearest neighbor graph on random geometric points has a high probability to be connected even with a very small number of neighbors (*d*) [31]. To empirically test this as well as to find the range of *d *for typical microarray data, we randomly generated a data set with 1000 genes and various dimensions (conditions) using Gaussian distribution. We then constructed a rank-based co-expression network using different values of *d *and measured the number of disconnected components in the resulting network. Remarkably, we find that for data of dimension > 10, a nearest neighbor graph with 1000 nodes is almost always fully connected with *d *= 2 neighbors. Even for data of smaller dimensions, the graph can be connected with at most 4 neighbors (Figure 2). The results do not vary significantly when the number of genes or the type of distribution is changed. In the next subsection, we also show that the yeast gene co-expression network is connected with *d *= 2. In practice we find a value of *d *between 3 and 5 is sufficient for most cases. This simple network construction method can also be combined with other strategies that were developed for value-based networks. For example, the raw similarity values can be refined by considering local neighborhood or shortest path information before rank transformation [16]; when selecting edges according to ranks, a threshold based on raw similarity values may be imposed simultaneously to ensure confidence in the edges being created. Ideally, methods can also be developed to automatically select the optimal *d*, as in [8]. The rank-based method can also be applied to construct networks of other entities, as long as a similarity measure can be defined. One example is to construct a network of samples from microarray data, where the nodes are samples and the similarity between two samples can be measured by the Pearson correlation coefficient between their gene expression profiles. Later we will show an application of a sample co-expression network where each sample is a cell type.

#### Topology of yeast co-expression networks

Previous studies have analyzed the topologies of various networks, including biological and social networks, and suggested three common topological properties: scale-free, small-world, and hierarchically modular [26,27,32-34]. Although debate exists [35,36], it is generally believed that these properties may be related to the robustness and stability of the underlying systems [26,27,32-34]. For example, a small-world network has a small diameter and a large clustering coefficient (see Methods), which is believed to be related to an efficient and controlled flow of information [26,34]. In a scale-free network, the probability for a node to have *k *edges follows a power-law distribution, i.e. *P*(*k*) = *c *× *k*^-*γ*^. The implication of the scale-free property is that a few nodes in the network are highly connected, acting as hubs, while most nodes have low degrees. Scale-free networks are believed to be robust to random failures, but vulnerable to deliberate attacks [27,32]. In comparison, in a random network (specifically, an Erdos-Renyi random network [26]), connections are spread almost uniformly across all nodes [26,34]. Furthermore, although a random network may also have a small diameter, it usually has a near zero clustering coefficient [26,34]. Several studies have analyzed the value-based gene co-expression networks, and reported some interesting but controversial results [11-13,25]. Here we analyze the topologies of both the rank-based and the value-based networks, and compare with previous results.

We obtained a set of yeast gene expression data measured in 173 different time points under various stress conditions [37], and selected 3000 genes that showed the highest variations. We constructed four gene co-expression networks using the rank-based method with *d *= 2, 3, 4 and 5, respectively. For each rank-based network, we constructed two random networks as follows. First, we randomly permuted the expression data of each gene independently, and constructed a rank-based network using the permuted data. Second, we randomly rewired the connections in a true rank-based network, but preserved the degree for every node [34]. For comparison, we also constructed four value-based networks, using the Pearson correlation coefficient as a similarity measure. The thresholds were chosen such that the average degrees are 10, 30, 50, and 100, respectively, in the resulting networks. Similar to the rank-based networks, we obtained two random networks for each true value-based network, one constructed from randomly permuted data and the other by randomly rewiring the true network.

Table 1 lists some statistics of these networks. In the rank-based networks, almost all genes are linked to the largest component with *d *as small as 2. Furthermore, compared to both the randomly rewired networks and the networks constructed from randomly permuted data, the true rank-based co-expression networks have slightly larger average path lengths and diameters, but much larger clustering coefficients, indicating that the rank-based co-expression networks have the small-world property. In contrast, the true value-based co-expression networks contain many singletons. For example, with a Pearson correlation coefficient threshold of 0.69, about 900 genes are singletons, even though the average node degree is much higher than in the rank-based networks. Furthermore, although the value-based networks have high clustering coefficients, their randomly rewired counterparts have almost similarly high clustering coefficients. This observation suggests that the high clustering coefficient of the value-based networks is partially because their non-singleton nodes are almost completely connected, in which case the structure cannot be destroyed by any random rewiring.

**Table 1:** Statistics of yeast co-expression networks

	Rank-based networks											
	
	Network from real data				Network from permuted data				Randomly rewired network			
*d*	2	3	4	5	2	3	4	5	-	-	-	-
Number of singletons	0	0	0	0	0	0	0	0	0	0	0	0
Size of largest component	2971	3000	3000	3000	3000	3000	3000	3000	3000	3000	3000	3000
Average degree	3.6	5.4	7.2	9.0	2.8	4.0	5.2	6.3	3.6	5.4	7.2	9.0
Largest degree	22	35	49	57	10	13	14	17	22	35	49	57
Clustering coefficient	0.17	0.19	0.21	0.22	0.01	0.02	0.02	0.02	0.001	0.002	0.004	0.005
Average path length	8.8	6.8	5.9	5.4	9.6	6.4	5.3	4.7	6.0	4.6	4.1	3.7
Diameter	19	19	14	11	17	10	8	7	12	8	7	6

	**Value-based networks**											
	
	**Network from real data**				**Network from permuted data**				**Randomly rewired network**			

Correlation cutoff	0.85	0.79	0.76	0.69	0.21	0.18	0.16	0.14	-	-	-	-
Number of singletons	2163	1586	1311	893	0	0	0	0	2163	1586	1311	893
Size of largest component	486	756	1609	2055	3000	3000	3000	3000	835	1412	1689	2107
Average degree	10(37)	30(64)	50(107)	100(142)	10	30	50	100	10	30	50	100
Largest degree	211	340	418	581	24	55	79	136	211	340	418	581
Clustering coefficient	0.52	0.59	0.62	0.64	0.03	0.04	0.05	0.07	0.31	0.34	0.34	0.34
Average path length	2.6	2.6	6.0	4.8	3.8	2.7	2.4	2.0	2.5	2.4	2.3	2.2
Diameter	9	9	18	13	6	4	3	3	5	5	5	5

Numbers in parentheses are for non-singleton nodes only.

**Figure 3:**
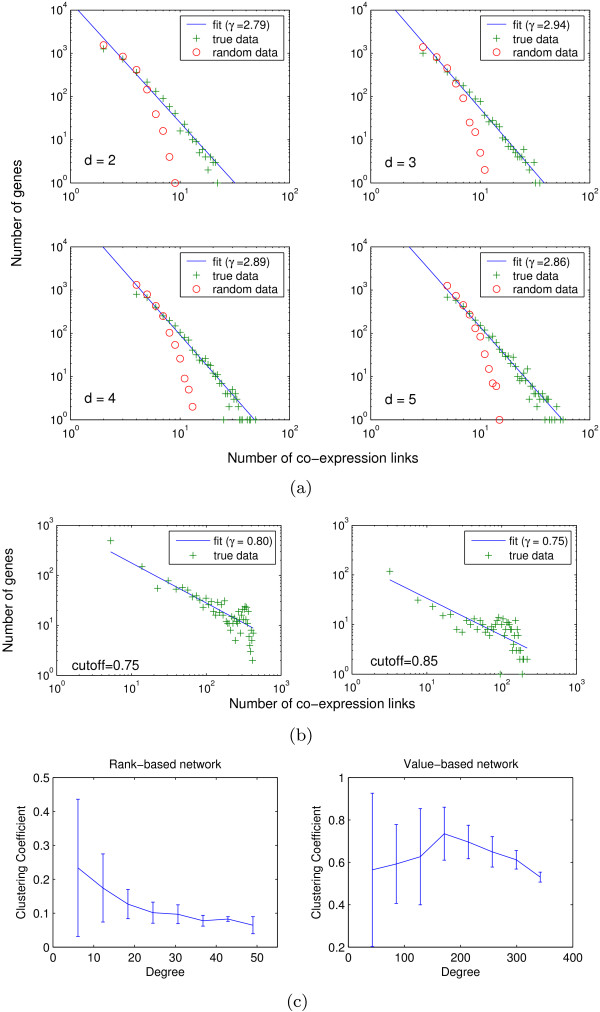
**Topological properties of co-expression networks**. (a) Degree distribution of rank-based co-expression networks. (b) Degree distribution of value-based co-expression networks. (c) Relationship between clustering coefficient and degree in rank-based and value-based co-expression networks.

Figure 3(a) and 3(b) shows the degree distributions of these networks. As indicated by a linear relationship in the log-log plot, the rank-based networks constructed from the real data exhibit a power-law degree distribution for all the *d *values considered. This suggests that an overall scale-free topology is a fairly robust feature of the co-expression networks. In contrast, the networks constructed from randomly permuted gene expression data contain significantly fewer high-degree nodes, and exhibit exponential degree distributions. The value-based networks appear to follow power-law degree distributions as well; however, they have a much larger number of high degree nodes than the rank-based networks.

To quantify the difference between the degree distributions of the value-based and rank-based networks, we fitted a power-law function for the degree distribution of each network to determine its *γ *parameter. The values of *γ *in the rank-based networks are consistently between two and three. This is typical in many biological networks such as PPI networks and metabolic networks, as well as in real-world social and technology networks [26,34]. In comparison, the *γ *values in the value-based networks are below one (Figure 3b). Theoretically, it is known that a scale-free network with *γ *< 2 has no finite mean degree when its size grows to infinity, and is dominated by nodes with large degrees [26]. Therefore, small values of *γ *for co-expression networks were reported in several previous studies as a significant difference between the co-expression networks and other biological networks [15,17]. Our results suggest that this difference may simply be an artifact of the network construction method. Consider that genes in some modules are strongly co-expressed with one another, while genes in some other modules are weakly co-expressed. Using the value-based method, when the similarity cutoff is gradually decreased, the genes within the strongly co-expressed modules will be first connected, up to a point that they are almost completely connected, before any gene in the weakly co-expressed modules can be connected to their within-module partners. As a result, the co-expression network will have many genes with large degrees, resulting in a small slope in the log-log plot. In contrast, with the rank-based method, genes in both strongly and weakly co-expressed modules can be connected, as essentially a different similarity threshold is used for each gene. Therefore, rank-based networks can usually capture the topology of both strongly and weakly co-expressed modules, while value-based networks are often dominated by the strongly co-expressed modules.

Moreover, previous studies have reported that gene co-expression networks lack the hierarchically modular property [11,12]. This property is characterized by a reciprocal relationship between a node's degree and its clustering coefficient [33]. Again, we have found that this claim only applies to the value-based networks. As shown in Figure 3(c), there is a clear reciprocal relationship between the node degree and node clustering coefficient in the rank-based networks, when compared to the value-based networks. This suggests that gene co-expression network can also have hierarchical structures.

Together, these experiments show that the rank-based co-expression networks have all the common topological properties of many other biological networks, while the value-based networks seem to differ significantly. Although these do not necessarily prove that rank-based networks are biologically more meaningful than value-based networks, the former seems to be able to capture the underlying topological structures better.

### Module discovery and analysis in gene co-expression networks

Gene co-expression networks with thousands of nodes are difficult to visualize and comprehend. A useful strategy for analyzing such a network is to partition it into subnetworks, where the nodes within each subnetwork are relatively densely connected to one another but have fewer connections to the other subnetworks. In gene co-expression networks, such subnetworks can be considered as candidates of functional modules, as genes within each subnetwork are mutually co-expressed, while co-expression between genes in different subnetworks are sparse. Many graph partitioning algorithms have been developed in computer science [38]. Similar to clustering, one major difficulty in graph partitioning is to determine the number of partitions. Some methods do not require this to be explicitly determined in advance, but require other parameters, which are also difficult to obtain. For example, MCL, one of the best graph partitioning algorithms, requires an inflation parameter, and setting the parameter to different values may result in very different results [29].

To address this difficulty, we introduce an algorithm that we have developed recently for identifying "communities" in arbitrary networks [28]. The main motivation for the algorithm is that each "community", or a subnetwork, must contain more intra-community edges than would be expected by chance if the connections were random. With this motivation, we developed an algorithm to optimize an objective function called modularity, which is precisely defined as the percentage of intra-community edges minus the random expectation (see Methods). The algorithm, named *Qcut*, has been shown to be effective in finding statistically significant and practically interesting graph partitions in many synthetic networks, social networks, and biological networks, without any user-tunable parameters, and has outperformed the existing algorithms based on similar motivations [28].

We evaluate the performance of *Qcut *on gene co-expression networks in several ways. We first use synthetic microarray data where the true modular structure is known, so that we can directly measure the accuracy. We then use two real microarray data sets to evaluate the overall biological significance of the identified gene modules, with two different metrics. The first metric is a commonly used approach based on the enrichment of specific Gene Ontology terms in the modules, which may be biased by the number of modules and module sizes. The second is a new metric that we introduced based on the idea of reference networks, which can be obtained from a variety of sources, such as gene annotations or protein-protein interaction networks (See methods).

#### Evaluation using synthetic microarray data

To objectively evaluate the accuracy of the modules detected by *Qcut*, we tested it on a large collection of synthetic gene expression data. The data sets, available at http://www.biostat.pitt.edu/bioinfo/publication.htm, were used to evaluate many clustering algorithms in a previous study [39]. Each data set contains simulated expression data of approximately 600 genes under 50 conditions. Each gene was pre-assigned to one of fifteen clusters, and the genes in the same cluster had their expression profiles generated from a common log normal distribution. Gaussian noises were then added to the data set to simulate experimental noises. A higher level of Gaussian noise generally makes the data more difficult to cluster. Since the correct clusters are known, we used a well-known metric called the adjusted Rand Index to measure the accuracy of *Qcut *(see Methods) [40].

**Figure 4:**
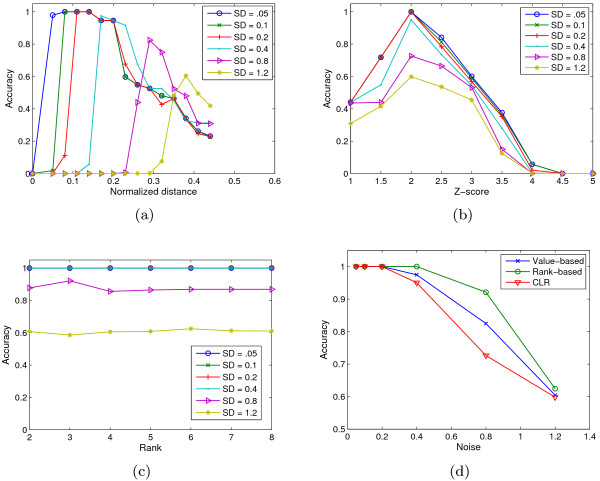
**Effects of network construction methods on the clustering accuracy of Qcut**. (a) Clustering accuracy on value-based networks, as a function of the distance cutoffs. (b) Clustering accuracy on CLR co-expression networks, as a function of the Z-score cutoffs. (c) Clustering accuracy on rank-based networks, as a function of the rank cutoffs. (d) Best clustering accuracy on the three types of networks, constructed with the optimal cutoffs. In all four plots, each data point is an average over the results of 100 synthetic microarray data sets.

**Figure 5:**
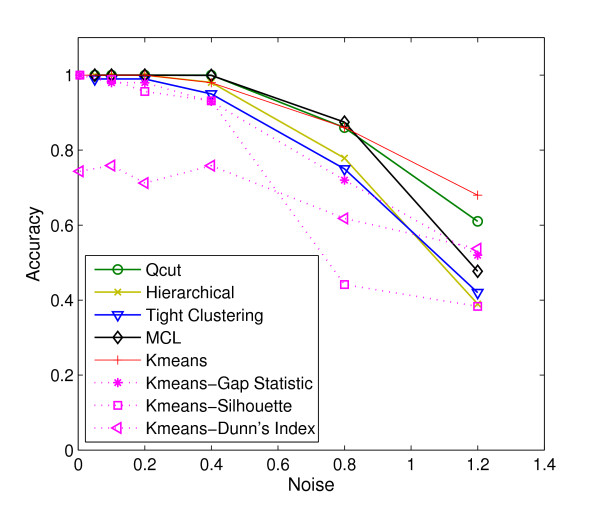
**Comparison of different clustering methods using synthetic microarray data**. *Qcut *and *MCL *are applied to rank-based networks constructed with *d *= 4. Each data point in the plot is an average over 100 synthetic microarray data sets.

We first compared the accuracy of *Qcut *on co-expression networks constructed by three methods: value-based, rank-based, and CLR [19]. We used Euclidean distance as the basis to measure the dissimilarity between two genes. For the value-based method, we normalized the distance to be between 0 and 1, and constructed a series of co-expression networks for each data set using different threshold values. As shown in Figure 4(a), the threshold that results in the best clustering accuracy varies for different data set. For more noisy data set a larger threshold value is needed, which suggests that choosing a right threshold is critical for the value-based method. The CLR method, in contrast, by converting the raw distances to z-scores, effectively removed such dependency and the best clustering accuracy is achieved at the same z-score equal to 2, corresponding to a p-value 0.05, for all data sets (Figure 4b). Interestingly, for the rank-based method, the clustering accuracy is almost invariant for rank cutoffs between 2 and 8 (Figure 4c). Figure 4(d) shows the best accuracy that can be achieved on the three types of networks. As can be seen, the rank-based networks clearly have the highest accuracy for intermediate levels of noises (SD = 0.4 or 0.8). For data with lower noises, all three methods resulted in perfect accuracy, and for data set with the highest level of noise (SD = 1.2), all three methods converges to about the same accuracy. Next we compared the clustering accuracy of *Qcut *on rank-based networks with several widely-used clustering algorithms including *k*-means clustering, hierarchical clustering [1], and tight clustering [39], applied directly to the gene expression data without deriving co-expression networks. In this test, *Qcut *was applied to rank-based co-expression networks constructed using *d *values equal to 4. In addition, we also tested one of the best graph partitioning algorithms called the Markov Clustering algorithm (MCL) [29], which is applied to rank-based networks as well. Since the results of MCL depend heavily on the choice of an inflation parameter (*I*), we applied MCL to the rank-based networks constructed with *d *fixed at 4, but varied *I *from 1.3 to 1.7, with an increment of 0.1, and took the best clustering accuracy resulted from these parameters. We used the MATLAB (the MathWorks Inc.) implementation of the *k*-means and hierarchical clustering algorithms. *k*-means clustering was run with *k *equal to 15, and was repeated 50 times for each data set to obtain the best results. The hierarchical clustering was performed using average linkage, and the final cluster tree was cut at an appropriate depth to generate 15 clusters. The accuracies of tight clustering were directly obtained from the original study that was done on the same data set [39]. Our evaluation results show that, even without any parameter tuning, *Qcut *outperformed the competing algorithms in identifying the true modular structures embedded within the synthetic microarray data. As shown in Figure 5, the clustering accuracy of *Qcut *is clearly better than that of the hierarchical clustering and tight clustering. The accuracy of *Qcut *is similar to *k*-means, except for the data sets of the highest level of noise. The synthetic data set with the highest level of noise may represent an extreme case in practice, as many of the clusters in this data set are not distinguishable visually (Figure S1 in Additional File 1). However, *k*-means achieved this accuracy with the number of clusters given explicitly, while *Qcut *did not have this information at all. In these synthetic data sets, the number of clusters is the single most important parameter and *k*-means is expected to work well when that is known. We tried to combine *k*-means with several popular methods to automatically determine the number of clusters, including the gap statistic [41], Silhouette [42], and the Dunn's Index [43]. Our results suggest that if the values of *k *are automatically determined, *k*-means performs much poorer than our method, especially for data sets with SD ≥ 0.4 (Figure 5). The results of MCL are two-fold. On one hand, when an appropriate inflation parameter is chosen (*I *= 1.5 in this experiment), MCL has an accuracy similar to that of *Qcut*, except for the data set with the highest noises, indicating a superior performance of graph-based algorithms in general. On the other hand, the accuracy of MCL depends on the choice of the inflation parameter, and may be much lower than that of clustering algorithms if a suboptimal inflation parameter is used (data not shown).

#### Functional modules in a yeast gene co-expression network

To evaluate the performance of our algorithm on real biological data, we applied *Qcut *to cluster the four rank-based yeast co-expression networks constructed in the previous subsection. The best numbers of clusters suggested by *Qcut *for the four networks are 24, 20, 12 and 12, respectively. As shown on synthetic data, when the number of clusters is known, the clustering algorithms that explicitly use this information, such as *k*-means, perform better than those without this information, such as the hierarchical clustering. Therefore, here we compared the performance of *Qcut *to *k*-means and two other popular clustering algorithms, namely self-organizing maps (SOM) [44] and spectral clustering [45], both of which require the number of clusters to be given. Applying the value-based method to the yeast data set, as we showed in Figure 1, the network either contains too many singletons, or are too densely connected to be partitioned by *Qcut *(or any other graph algorithms). Therefore, we did not evaluate its accuracy on the real data set. Interestingly CLR had similar behavior as the value-based method on the yeast data set, indicating that it is partially value-based.

We used the SOM implementation in the microarray software suite TM4 from TIGR, and implemented our own version of spectral clustering. K-means, SOM and spectral clustering were applied directly to the expression data, using Pearson correlation coefficient as the similarity metric. We obtained 24, 20, 12 and 9 clusters using each of the three competing algorithms. To test if the competing algorithms may give a better result with a different setting of the number of clusters, we also applied the spectral clustering with the number of clusters *k *equal to 5, 6, ..., 25. SOM was executed on 4 × 6, 4 × 5, 3 × 4, and 3 × 3 grids to produce the desired number of clusters. Because *Qcut *identified 12 clusters on both the *d *= 4 and *d *= 5 networks, we compared the 12 clusters identified from the *d *= 5 network by *Qcut *with the 9 clusters identified by the competing algorithms to avoid redundant comparison. Another reason for this comparison is that *Qcut *often produces a few small clusters, while the clusters of the competing algorithms are relatively uniform in size. Therefore, the "effective" number of clusters is smaller for *Qcut *than for other algorithms, so we used this comparison to compensate for the differences in cluster sizes.

**Figure 6:**
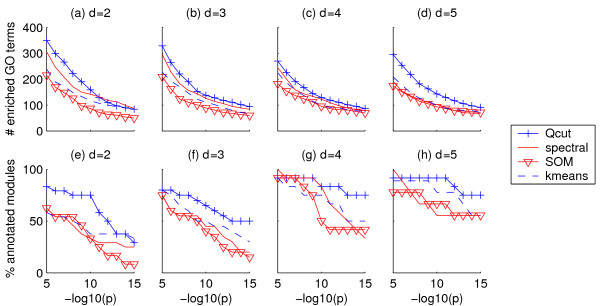
**Enrichment of GO terms in yeast co-expression networks**. Vertical axes in (a)-(d): number of GO terms enriched in the clusters. Vertical axes in (e)-(h): percentage of clusters that are enriched with at least one GO term. Horizontal axes: *p*-value cutoff to consider a GO term enriched.

To validate the biological significance of the clusters, we first counted the number of Gene Ontology (GO) terms enriched in the clusters and the percentage of clusters that had at least one enriched GO term, at various significance levels. As shown in Figure 6, the clusters identified by *Qcut *contain more enriched GO terms than the competing algorithms for most of the *p*-value cutoffs (Figure 6(a)-(d)). Furthermore, the percentage of clusters containing at least one enriched GO term is also higher for *Qcut *than for the other algorithms (Figure 6(e)-(h)).

**Figure 7:**
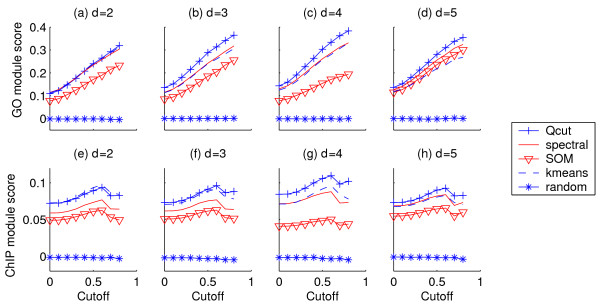
**Yeast gene co-expression network module scores based on reference networks**. Reference networks are derived from GO annotations (a-d) and ChIP-chip data (e-h). Horizontal axes: edge weight cutoff for the reference networks.

Second, as the above measurement may be biased by the number of clusters or the cluster size distributions, we compared these algorithms using a novel metric based on three reference networks that capture different functional interactions between genes: a co-annotation network based on GO terms of biological processes, a co-regulation network based on ChIP-chip data, and a PPI network (see Methods). While the PPI network has unweighted edges, the GO-based and ChIP-based reference networks have weighted edges. We discretized the GO-based and ChIP-based reference networks using a series of weight cutoffs. We then scored the gene modules identified by each algorithm using these reference networks (see Methods). As shown in Figure 7 and Figure S2 in Additional File 1, the gene modules identified by *Qcut *always had the highest score, using all three types of reference networks. The spectral clustering algorithm performed better than the other clustering algorithms. As *Qcut *and the spectral clustering algorithm share some similar spirit in capturing the topology embedded in a data matrix, this result seems to suggest that topological features are important for achieving good clustering results on gene expression data. We also randomly shuffed the gene modules identified by *Qcut *while preserving the sizes of the modules, and scored the random clusters using the reference networks. The scores for the random modules are very close to zero in all cases (Figure 7 and Figure S2 in Additional File 1), indicating that the results obtained by

#### Qcut and the clustering algorithms are not due to chance

The modules in the *d *= 2 co-expression network have the worst scores according to all three reference networks (Figure 7 and Figure S2 in Additional File 1), indicating that the *d *= 2 network might be too sparse to capture all functional relationships. The *d *= 4 co-expression network has the highest scores according to all three reference networks, while the networks with *d *= 3 or 5 give slightly worse results. As stated above, to test if the competing algorithms may give a better result with a different setting of the number of clusters, we applied the spectral clustering, which has a better accuracy than the other two algorithms, with the number of clusters *k *equal to 5, 6, ..., 25. We scored the gene modules using the GO-based reference network at a weight cutoff of 0.8. As shown in Figure S3 in Additional File 1, the best module score achieved by spectral clustering is 0.323 (at *k *= 13), which is significantly lower than the module score by *Qcut *(*Q *= 0.384). This shows that our evaluation results have not been biased by the number of clusters.

In addition, we combined *k*-means with gap-statistic [41], which estimated the optimal number of clusters to be 6. We also tested SOTA [46], a hierarchical version of SOM that can automatically determine the number of clusters. SOTA returned 11 clusters, a number similar to our optimal number. However, only 6 of the 11 clusters had significantly enriched functions, and the most significant function had a *p*-value 10^-49^, as compared to 10^-106 ^in our method (Table 2 and Table S1 in Additional File 1).

**Table 2:** Functional modules in a yeast co-expression network

Cluster	Size	**Category**^1^	Term	Count	**Enrichment**^2^	P-value
1	361	BP	protein catabolism	32	4.2	2.0E-12
		BP	protein folding	21	5.9	1.6E-11

2	498	BP	ribosome biogenesis	133	9.2	2.0E-106

3	18	BP	chromatin assembly or disassembly	9	36.4	5.3E-13
		TF	HIR2	6	129.8	2.3E-12
		TF	HIR1	6	62.9	3.0E-10
		TF	HIR3	6	57.7	5.3E-10

4	25	BP	telomerase-independent telomere maintenance	4	82.3	1.1E-07
		BP	biological process unknown	16	2.9	7.6E-06
		TF	GAT3	13	56.8	3.5E-21
		TF	YAP5	15	43.5	5.8E-17
		TF	PDR1	9	25.8	3.1E-11
		TF	MSN4	8	35.0	3.8E-11

5	422	BP	spore wall assembly	16	7.0	1.6E-10
		BP	biological process unknown	138	1.5	1.2E-07
		TF	NRG1	21	4.2	1.4E-08
		TF	SUM1	16	3.9	2.3E-06
		TF	PHD1	15	3.4	3.2E-05

6	99	-	-	-	-	-

7	463	BP	carbohydrate metabolism	41	2.9	4.6E-10
		BP	biological process unknown	178	1.7	9.5E-17
		BP	response to stimulus	62	1.7	2.0E-05
		TF	UME6	25	2.5	2.6E-05
		TF	NRG1	15	2.8	3.6E-04

8	108	BP	nitrogen compound metabolism	25	7.0	5.2E-15
		TF	MET31	4	9.6	8.0E-04
		TF	MET32	5	5.7	2.1E-03

9	192	BP	generation of precursor metabolites and energy	50	8.2	7.5E-33
		TF	HAP4	22	9.2	5.1E-16

10	22	BP	Ty element transposition	17	58.6	6.2E-29
		TF	SUM1	4	18.9	5.8E-05

11	604	BP	carboxylic acid metabolism	76	3.0	2.4E-19
		BP	cell organization and biogenesis	212	1.6	3.7E-15
		TF	SWI6	45	2.9	3.6E-11
		TF	SWI4	44	2.8	2.7E-10
		TF	FKH2	35	3.0	4.7E-09
		TF	MBP1	36	2.8	1.9E-08
		TF	STE12	22	3.6	7.9E-08
		TF	NDD1	30	2.9	1.1E-07
		TF	FKH1	34	2.5	9.6E-07
		TF	MCM1	22	2.9	3.9E-06

12	186	BP	protein biosynthesis	131	6.4	6.4E-85
		TF	FHL1	96	17.1	3.3E-105
		TF	RAP1	58	11.5	2.2E-48

^1 ^For each cluster, significantly enriched biological process GO terms (BP) or binding of transcription factors (TF) are counted.

^2^Fold of enrichment is calculated as: .

Table 2 summarizes the modules identified by *Qcut *from the *d *= 4 network. For each module, we show the most significantly enriched GO biological process terms and the transcription factors whose targets are significantly enriched in the module (see Methods). As shown, most modules contain highly coherent functional terms, and are co-regulated by common transcription factors. For example, the majority of genes in module 12 are involved in protein biosynthesis (*p *= 10^-85^), and can be bound by FHL1 (*p *= 10^-105^) and RAP1 (*p *= 10^-48^), both of which are known to be involved in rRNA processing and regulating ribosomal proteins [47]. Module 9 is significantly enriched with genes that are involved in generation of precursor metabolites and energy (*p *= 10^-33^), and can be bound by HAP4 (*p *= 10^-16^), a TF regulating carbohydrate metabolism [47]. Module 2 contains almost two-thirds of the ribosome biogenesis genes (*p *= 10^-106^), but no TFs were found to bind to this set of genes specifically. Module 11 is enriched with genes that can be bound by eight different TFs. Interestingly, all eight TFs are known cell-cycle regulators [47]. Several small modules correspond to specific functional groups. For example, 17 of the 22 genes in module 10 are involved in Ty element transposition (*p *= 10^-29^). Half of the 18 genes in module 3 are related to chromatin assembly or disassembly (*p *= 10^-13^); six of them are regulated by transcription factors HIR1/2/3, which are known to be involved in the transcription of histone genes [47]. Modules 5 and 7 contain both a large fraction of genes with unknown functions and groups of genes with significantly enriched common functions or common TFs. It is possible that these uncharacterized genes also have the functions that are enriched in the module.

**Figure 8:**
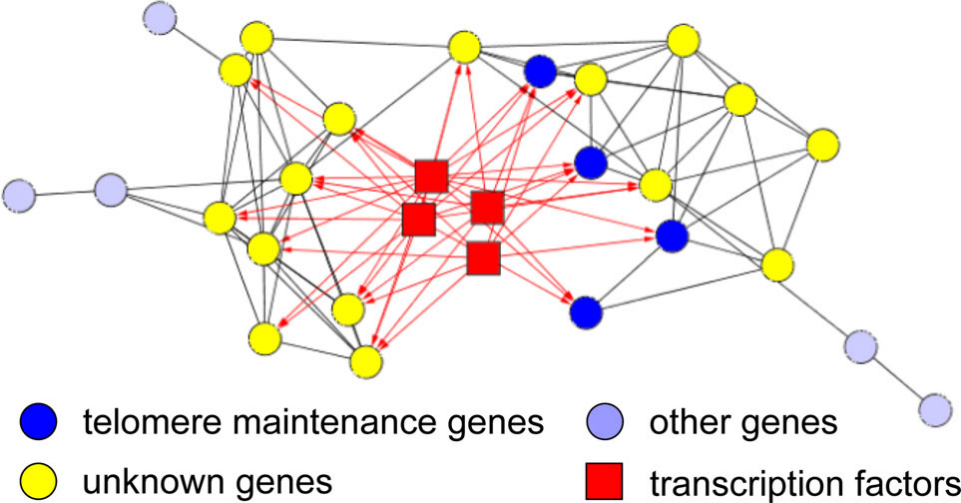
**A network of co-expressed and co-regulated genes with functions in telomerase maintenance**. Each directed edge pointing from a TF to a gene represents a protein-DNA interaction. All other edges represent co-expression relationships.

We also found a very interesting small module that may deserve further investigation. Among the 25 genes in module 4, four genes have a common function in telomere maintenance (*p *= 10^-7^). While the majority (16) of the remaining genes encode hypothetic proteins and have unknown functions (*p *= 10^-6^), a careful inspection showed that all four characterized genes and five of the 16 uncharacterized ones are located near telomeric regions [47]. Moreover, a significant number of genes in this module are regulated by four common transcription factors: GAT3 (*p *= 10^-21^), YAP5 (*p *= 10^-17^), PDR1 (*p *= 10^-11^), and MSN4 (*p *= 10^-11^) (Figure 8 and Table 2). Our results suggest that these uncharacterized genes as well as the four transcription factors are very likely to be involved in the function or maintenance of telomere, which has not been reported in the literature.

#### Functional modules in an Arabidopsis gene co-expression network

To test our method on high organisms, we applied it to a set of Arabidopsis gene expression data downloaded from the AtGenExpress database http://www.uni-tuebingen.de/plantphys/AFGN/atgenex.htm. We studied the cold stress response, for which the dataset contains the expression of 22 k Arabidopsis genes in the root and shoot tissues in six time points following cold stress treatment and under the normal condition. We selected 2545 genes that are up-or down-regulated by at least five-folds in at least one of the six time points in either tissue. We then constructed a co-expression network by connecting each gene to its top three correlated genes (i.e. *d *= 3), resulting in 5838 co-expression links in total.

**Figure 9:**
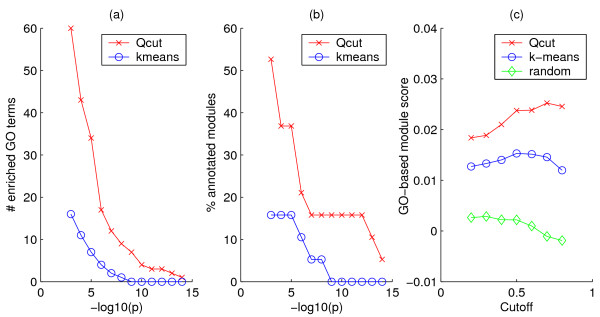
**Enrichment of GO terms in the Arabidopsis co-expression network**. (a) Number of enriched GO terms; (b) Percentage of clusters with at least one enriched GO term; (c) Gene module scores measured by the GO-based reference networks. Horizontal axes in (a) and (b) are *p*-value cutoffs on GO term enrichment. The horizontal axis in (c) corresponds to the edge weight cutoffs for reference networks.

Our clustering algorithm partitioned the network into 19 modules, with a *Q *value of 0.81, indicating a strong modular structure. Similar to the previous experiment, we first examined the GO terms enriched in the clusters at various significance levels, and compared them with the results of the *k*-means algorithm with *k *= 19. As shown in Figure 9(a) and 9(b), the modules identified by our method contain significantly more enriched GO terms than that identified by *k*-means, and GO terms are enriched in more modules in our method than in *k*-means. Furthermore, our method achieved significantly higher module scores in the GO-based reference networks than the *k*-means algorithm (Fig 9(c)). Table S2 in Additional File 1 lists the most enriched functional categories for each module. Several modules are enriched with functions that are known to be related to cold stress responses, e.g. modules 7 (photosynthesis, *p *= 10^-16^), 11 (circadian rhythm, *p *= 10^-5^), 14 (response to heat, *p *= 10^-15^), 15 (antiporter activity, *p *= 10^-6^) and 18 (lipid binding, *p *= 10^-8^). Due to the scarcity of functional annotations in Arabidopsis, the enrichment *p*-values as well as the module scores of the gene modules are less significant in Arabidopsis than in yeast, which is to be expected.

### Cancer subtype classification from sample co-expression networks

In this subsection, we show that our co-expression network analysis method can also be applied to identify sample modules. Conceptually, there is no difference in constructing and analyzing gene or sample co-expression networks: in the latter we treat each sample as a network node, and connect two samples when their expression profiles are similar according to some similarity measure. In practice, however, sample co-expression networks are usually smaller than gene co-expression networks, but the edges in sample co-expression networks may be computed from very high-dimensional data, since each sample is described by thousands of genes. It is therefore interesting to see whether the same network construction and module detection methods can be applied here without much tuning of the parameter, i.e. the value of *d*. As an application of our approach, we applied our method towards an automatic classification of tumor cells.

An accurate classification of tumor cells is crucial for effective therapy [48]. Traditionally, tumors have been classified by their morphologic appearance, which is, unfortunately, often very subjective. Furthermore, tumors with similar histological features may respond very differently to chemotherapy [30]. To address this problem, a promising alternative or complementary strategy is to classify tumors based on their genetic profiles, i.e. the activity of hundreds or thousands of genes that are involved in the disease. Most of the existing tumor classification approaches are based on supervised learning, such as support vector machines or decision trees, which aimed at identifying genetic features to distinguish two or more known tumor (sub-)types [49,50].

Here, we ask whether it is possible to automatically classify tumor samples in the absence of training data that provide known tumor (sub-)types or even the number of distinct (sub-)types. This unsupervised learning approach has a few advantages over supervised learning methods. First, the existing tumor classifications are based on histological features, which may be unreliable themselves. Second, using unsupervised learning, we may be able to discover novel tumor sub-types that have not been characterized by histological features previously. On the other hand, it is crucial to confirm whether the automatically discovered tumor (sub-)types are biologically meaningful and indeed provide useful information for understanding the disease mechanism or for improving its treatment.

In this study, we chose to focus on lymphoma, a family of tumors involving cells of the immune system. We obtained a data set containing the expression data of 4026 genes for 96 samples belonging to nine cell types, including three different types of tumors, namely, diffuse large B cell lymphoma (DLBCB), chronic lymphocytic leukemia (CLL), and follicular lymphoma (FL), as well as normal B and T cells at different stages of cell differentiation [30]. We constructed a rank-based network of the samples using Pearson correlation coefficient as the similarity measure and the value of *d *at 5. Edges with a Pearson correlation coefficient < 0.2 were removed to reduce false connections.

**Figure 10:**
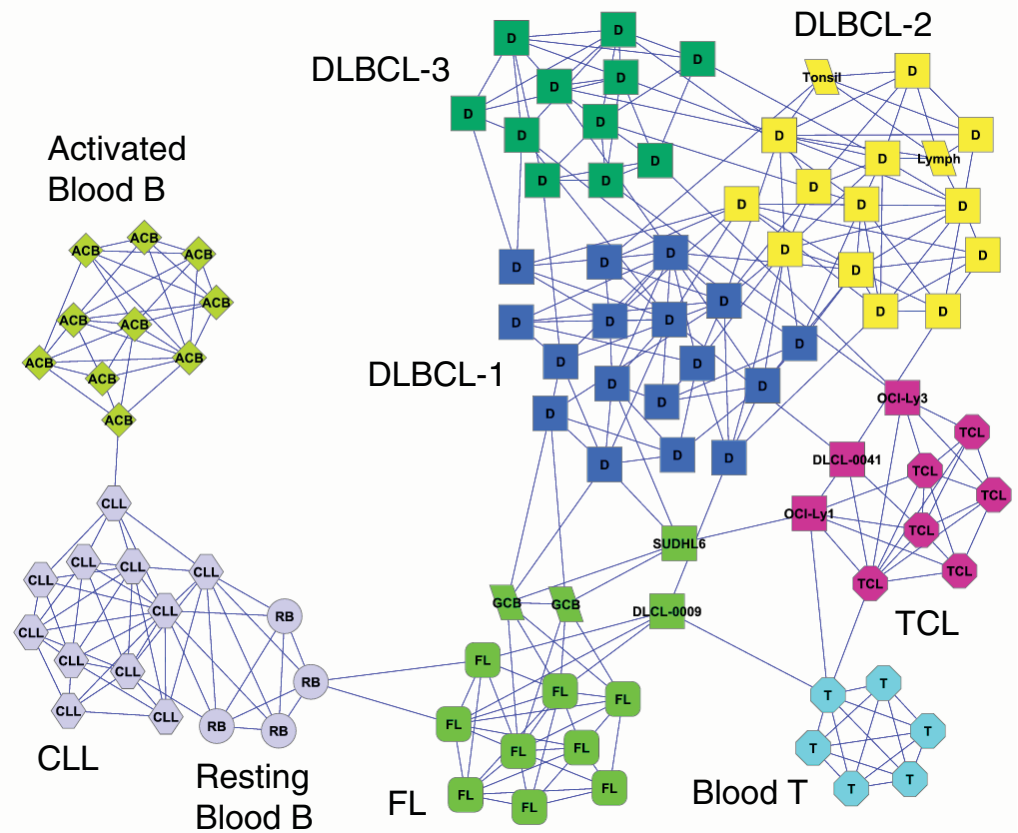
**A co-expression network of cancer cells**. Each cluster is shown with a different color. Each cell type is represented by a unique combination of the shape and text inside a node. Square nodes with D inside represent DLBCL cells. DLBCL outliers that were incorrectly classified are shown with their actual names inside square nodes. Abbreviations: TCL - transformed cell line; GCB - germinal centre B; DLBCB - diffuse large B cell lymphoma; CLL - chronic lymphocytic leukemia, FL - follicular lymphoma; ACB - activated blood B; RB - resting blood B.

Applying *Qcut *to the network, we identified eight modules. Remarkably, without any prior knowledge about the number of cell types in the samples, our algorithm automatically separated different cell types into different modules, with a few exceptions (Figure 10 and Table S3 in Additional File 1). Furthermore, the results are almost invariant when we varied *d *between 3 and 7, indicating a very stable modular structure among the samples. As shown in Figure 10, blood T cells and activated blood B cells are perfectly classified into their own modules. CLL and resting B cells are grouped into a single module, which is not surprising since the CLL has a very low proliferation rate, similar to resting B cells [30]. The two germinal centre B (GCB) cell samples are grouped with the FL cells and are far away from the activated B cells, which confirms the hypotheses that GCB represents a distinct stage of B cell differentiation, and that the FL arises from this stage of B-cell differentiation [30]. The transformed cell line module also contains three DLBCL cells. A closer inspection showed that two of the DLBCB samples in this module (OCI-Ly1 and OCI-Ly3) are laboratory-cultivated cell lines rather than samples from real patients (Figure 10), which may be the reason that they are grouped with the transferred cell lines. The Lymph node/tonsil cells are grouped with DLBCL as in previous studies [30].

Interestingly, the majority of the DLBCB samples are grouped into three modules (DLBCL-1, 2, and 3). It is well known that not all DLBCL tumors are equal: 40% of patients respond well to chemotherapy and have prolonged survival, while the others have a much shorter survival time after treatment [30]. Previous studies have suggested that DLBCL tumors can be categorized into two subtypes: GCB-like DLBCL and activated B-like DLBCL [30]. The GCB-like DLBCL shares gene signatures that distinguished germinal centre B cells from B cells in other stages, while the activated B-like DLBCL shared many gene signatures with normal lymph node and tonsil. On average, GCB-like DLBCL patients have a much higher survival rate than activated B-like DLBCL patients after comparable chemotherapy [30]. In our sample co-expression network, the GCB cells connect to DLBCL-1 cells while the tonsil and lymph node cells fall in the DLBCL-2 modules, suggesting that the two modules may correspond to the two well-known DLBCL subtypes. Indeed, the median survival durations for the patients in the DLBCL-1 and DLBCL-2 modules are 71 and 22 months, respectively. Furthermore, 11 out of 15 (73%) patients in the DLBCL-1 module lived more than five years after treatment, while only 2 out of 13 (15%) patients in the DLBCL-2 module survived that long. Even more interestingly, however, the DLBCL-3 module, which is apart from GCB and Tonsil/Lymph nodes, has the lowest survival rate overall. The median survival durations for this module is 12 months, and only and 1 out of 10 (10%) patients in this module survived more than five years after treatment. The lack of GCB-like or activated B-like signatures and the lower survival rate seems to suggest DLBCL-3 to be a separate subtype.

## Conclusions

In this paper, we presented a general co-expression network-based approach for the analysis of high-throughput gene expression data. We introduced a simple rank-based method to construct gene or sample co-expression networks, and an algorithm called *Qcut *to identify modules from the co-expression networks. We also introduced a novel metric to evaluate the functional significance of gene modules based on reference networks.

On synthetic data, we showed that our method can automatically discover the embedded modular structures and that our method significantly outperformed a number of competing algorithms. We applied our method to two real data sets, and showed that it can produce statistically more significant gene functional modules than conventional clustering methods such as *k*-means and self-organizing maps, evaluated by a number of criteria. Furthermore, our test on a sample co-expression network showed excellent results in separating different types or subtypes of tumor cells. All these were achieved without knowing the number of modules in advance.

We reported several interesting biological results, including some testable biological hypotheses. We showed that the rank-based co-expression networks have all the common topological properties that exist in other biological networks, challenging the previous results on the topology of gene co-expression networks. We discovered an interesting gene module in yeast suggesting that some uncharacterized genes and transcriptional regulators may be involved in maintaining telomere integrity. We also identified a module of tumor cells which seems to correspond to a new subtype that has not been identified before.

Although we have only demonstrated our method on gene expression data, it can be applied to other types of experimental data as well. The rank-based network construction method can be used to capture the relationships among other entities. The efficiency of our module detection method and its parameter-free feature make it well suited for identifying intrinsic structures in large-scale network data. Another advantage of our method is its flexibility in integrating different types of information. For example, co-expression networks and transcriptional regulatory networks can be easily integrated; the modules in those integrated networks will then represent co-expressed and co-regulated genes, as well as their regulators. Similarly, we would expect other types of information, such as protein-protein interaction, phylogeny and phenotypes, to be integrated to produce more biologically insightful results.

## Methods

### Network topological analysis

The topological properties of networks were analyzed using MATLAB (the MathWorks Inc.). For each network the number of nodes and number of edges was simply counted. A singleton is a node with zero connection. The average degree ⟨*k*⟩ was calculated as the average number of connections per node. The clustering coefficient of a network *C *was calculated as the average clustering coefficient of all of its non-singleton nodes using the formula: *C*_*i *_= 2*n*_*i*_/*k*_*i*_(*k*_*i *_- 1), where *n*_*i *_is the number of observed links connecting the *k*_*i *_neighbors of node *i *and *k*_*i*_(*k*_*i *_- 1)/2 is the total number of possible links. The average path length (⟨*l⟩*) was calculated as the average shortest path, or the smallest number of edges needed to connect two nodes, between any two reachable nodes in the network. The diameter was defined as the longest path length between any two reachable nodes in the network. Node degree distributions were plotted with the degree (*k*) on the x-axis and the number of nodes with this degree *f*(*k*) on the y-axis. Clustering coefficient against node degree *C*(*k*) distributions were plotted with the degree (*k*) on the x-axis and the average clustering coefficient ⟨*C*⟩ for all nodes with degree *k *on the y-axis.

### Module detection

Our module detection algorithm, *Qcut*, optimizes a modularity function *Q *to automatically determine the most appropriate number of modules in a network. The algorithm can handle networks of several thousands of nodes in a few minutes, much faster than most existing algorithms, and at the same time can often achieve better quality. We have extensively tested the algorithm on many synthetic networks and real-world networks with known community structures, as well as several real applications such as PPI networks and scientific collaboration networks. The results from these analyses show that our method is very efficient and effective. The detailed analysis and evaluation of the algorithm can be found in [28]. Here we briefly describe the key ideas in the algorithm.

The modularity function [51], *Q*, is defined as:(1)

where *Γ*_*k *_is a clustering that partitions the nodes in a graph into *k *groups, *e*_*ii *_is the fraction of edges with both nodes within cluster *i*, and *a*_*i *_is the fraction of edges with one or both nodes in cluster *i*. Intuitively, the *Q *function measures the percentage of edges fully contained within the clusters, subtracted by what one would expect if the edges were randomly placed. The value of *Q *is between -1 and 1; a larger *Q *value means stronger modular structures. If a partition gives no more within-cluster edges than expected by chance, *Q *≤ 0. For a trivial partitioning with a single cluster, *Q *= 0. It has been observed that most real-world networks have *Q *> 0.3 [26]. The *Q *function can also be extended to weighted networks straightforwardly by generalizing *e*_*ii *_and *a*_*i *_to edge weights, instead of number of edges.

Since the optimization of *Q *is NP-hard [52,53], we developed a heuristic procedure to achieve this goal. Given the adjacency matrix of a network *G*, we apply a standard spectral clustering algorithm [45] to search for the best 2-, 3, or 4-way partitioning that gives the highest *Q *value. This is recursively applied to partition each subnetwork until the overall *Q *value of the network does not increase. To further optimize *Q*, an efficient greedy search procedure is then repeatedly applied to look for the following possible operations: merging two modules, moving a node from one module to a different module, or further splitting a module. The procedure terminates when no operations can improve *Q*.

### Module evaluation

#### Comparing to known modular structures

To compare the modules identified by an algorithm to the true modules, we computed the adjusted Rand Index [40]. Given a set of objects *S *= *s*_1_, *s*_2_, ..., *s*_*n*_, let *X *= {*X*_1_, *X*_2_, ..., *X*_*M *_} and *Y *= {*Y*_1_, *Y*_2_, ..., *Y*_*N *_} represent the true and predicted partitions of the objects, where each object appears in *X *and *Y *exactly once. Let *n*_*ij *_be the number of common objects between *X_i _*and *Y_j_*. Also let *n*_*i*• _= ∑_*j*_*n*_*ij *_= |*X*_*i*_| be the size of *X*_*i*_, and *n*_*j*• _= ∑_*i *_*n*_*ij *_= |*Y*_*j*_| be the size of *Y*_*j*_. The adjusted Rand Index can be computed by:(2)

#### Statistical enrichment of GO terms and transcription factor targets

To assess the functional significance of gene modules, we first compute the enrichment of GO terms for the genes within each module. The statistical significance of GO term enrichment is measured by a cumulative hypergeometric test [54]. The *p*-values are adjusted by Bonferroni corrections for the multiple testing problem [54]. The search of enriched GO terms is performed with a computer program GO::TermFinder [55].

To compare different results with approximately the same number of modules, we count the number of GO terms enriched in the modules at a given significance level. Furthermore, to rule out the possibility that a single module may contain a very large number of enriched GO terms and therefore dominate the contribution from other modules, we also compute the percentage of modules that have at least one enriched GO term at a given significance level. It is worth noting that two sets of results cannot be compared by this method if they differ significantly in numbers of modules or modules size distributions, which may strongly affect the number of enriched GO terms. The results of the comparison may also depend on what *p*-value threshold is used.

The enrichment of transcription factor targets was determined similarly as in computing the enrichment of GO terms, and the binding data were from the large-scale ChIP-chip assay of 203 yeast transcription factors (TFs) under rich media conditions [56]. We only consider a binding as real if its *p*-value is less than 0.001, according to the original authors [56].

#### Reference network-based module evaluation

We propose a novel method for assessing the functional significance of gene modules. The basic idea is to introduce a functional reference network (discussed later), and compare the gene modules in a co-expression network with the structures of the reference networks. In such a reference network, genes are linked by edges that represent certain functional relationships between them, where the edges may be weighted according to the reliability or significance of the relationships. This network can be expected to have some modular structures as well. Since our purpose is to identify functional modules within a co-expression network, we would prefer a good partitioning of the gene co-expression network to represent a good partitioning of the reference network as well; i.e., genes within the same co-expression module should be connected by many high weight edges in the reference network, while genes in different co-expression modules should share less functions or be connected with low weight edges in the reference network. To quantify this, we force the reference network to be partitioned exactly the same way as the co-expression network, i.e., the group memberships of the nodes in the reference network are the same as that of the co-expression network. We then score the gene modules by the modularity of the reference network using Equation (1). The modularity score is between -1 and 1, with one meaning the co-expression network modules perfectly agree with the modular structure of the reference network. Since this measure is not biased by the number of modules or the module size distributions, it can be applied to compare arbitrary clustering results.

A reference network can be obtained from a variety of sources. First, available biological networks, such as PPI networks and genetic interaction networks, can be used as reference networks directly. A reference network can also be derived from other attributes of genes. In general, two genes can be connected if they possess some common attributes, given that the common attributes are related to co-expression. For example, co-expressed genes may participate in the same biological process or be regulated by a common TF. These types of information can be represented by a matrix, where each row is a gene, and each column is an attribute. To construct a reference network from the matrix, genes are treated as nodes, and an edge is drawn between two genes if they share at least one common attribute. Edges are weighted by some similarity measure of genes' attributes. To measure the similarity, we use a well-known function in machine learning that takes into account the significance of attributes [57]. For example, the Gene Ontology term GO:0009987 (cellular process), which is very close to the root of the Gene Ontology graph and has a large number of genes associated, is less informative and should be weighted less than the term GO:0045911 (positive regulation of DNA recombination).

Denote a gene-attribute matrix by *A *= (*a*_*ij*_), where *a*_*ij *_= 1 if gene *i *has attribute *j*, or 0 otherwise. *A *is transformed into a weighted matrix *W *= (*w*_*ij*_), where *w*_*ij *_= *a*_*ij *_× *idf_j_*. The weighting factor *idf_j_*, called the inverse document frequency (IDF) [57], is defined by *idf*_*j *_= log(*n*/∑_*i *_*a*_*ij*_), where *n *is the number of genes. With this transformation, the attributes that occur in many genes receive low weights in *W*. The edge weight between two genes is then measured by the cosine of their weighted attribute vectors:(3)

where *w*_*i*_. and *w*_*j*_. are the *i*-th and *j*-th rows of *W*, respectively. As expected, many genes may be connected with very low weights if they share some non-specific functions. We apply a weight cutoff to remove such edges. We have found, however, that the result is almost not affected by the use of different cutoff values, as shown in Results and Discussion.

We use three types of reference networks to evaluate clusters. The first is a network constructed from Gene Ontology biological process terms [58], with each term as an attribute. The ontology and annotation files for yeast and Arabidopsis genes are downloaded from http://www.geneontology.org/. To construct a reference network, we first convert the original annotation files to include complete annotations, i.e., if a gene is associated with a certain term, we also add all ancestors of the term into the gene's attribute list due to term inheritance. If two terms are associated with exactly the same set of genes, we remove one to avoid double counting. We also remove GO terms that are associated with more than 500 or less than 5 genes. The procedure results in 1034 and 438 GO terms for yeast and Arabidopsis, respectively. The second is a PPI network for budding yeast, downloaded from the BioGRID database [59]. We combined all physical interactions obtained from yeast two-hybrid or affinity purification-mass spectrometry experiments. The third network is a co-regulation network derived from the ChIP-chip data of 203 yeast transcription factors (TFs) under rich media conditions [56]. We treat each TF as an attribute, and construct a network with the procedure described above. We only consider a binding as real if its *p*-value is less than 0.001, according to the original authors [56].

## Authors' contributions

JR and WZ conceived of the research and designed the study. JR carried out most of the computational analysis. AKD participated in evaluating the methods using synthetic microarray data. JR wrote the paper and WZ helped with the manuscript preparation. All authors read and approved the final manuscript.

## Supplementary Material

Additional file 1 Figures S1-S3 and Tables S1-S3 Click here for file
