# Supplementary material for: A general co-expression network-based approach to gene expression analysis: comparison and applications
Source: BMC Syst Biol. 2010 Feb 2;4:8. doi: 10.1186/1752-0509-4-8 (PMC2829495; doi:10.1186/1752-0509-4-8)
Supplement: Additional file 1 — Figures S1-S3 and Tables S1-S3 [file 1752-0509-4-8-S1.PDF]

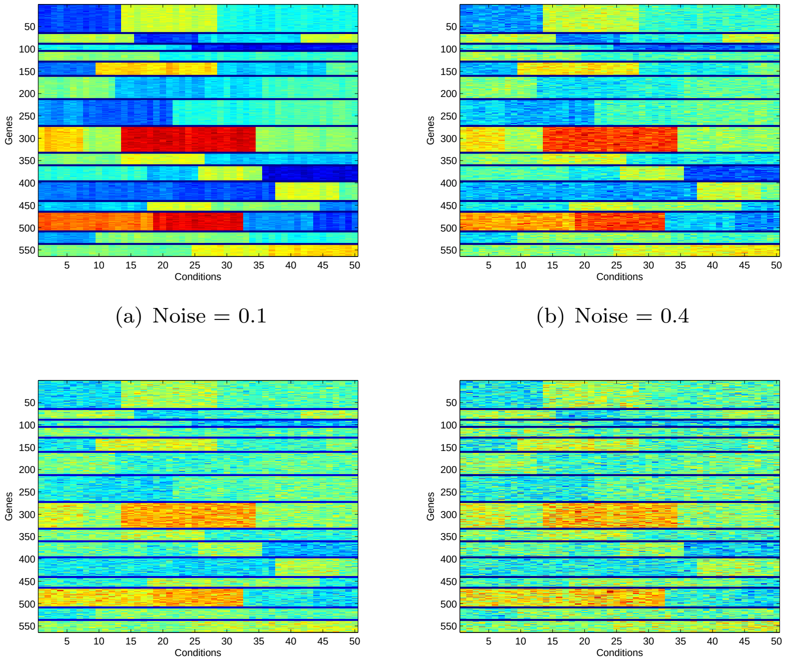

(a) Noise = 0.1

(b) Noise = 0.4

(c) Noise = 0.8

(d) Noise = 1.2

Figure S1: Heat map and modular structure of synthetic gene expression data.

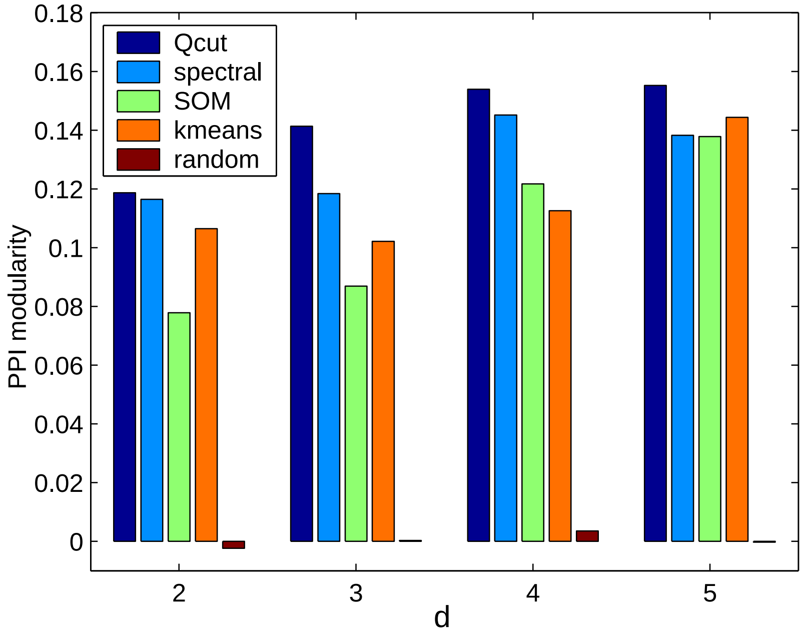

Figure S2: Yeast gene co-expression network module scores according to a PPI-based reference network.

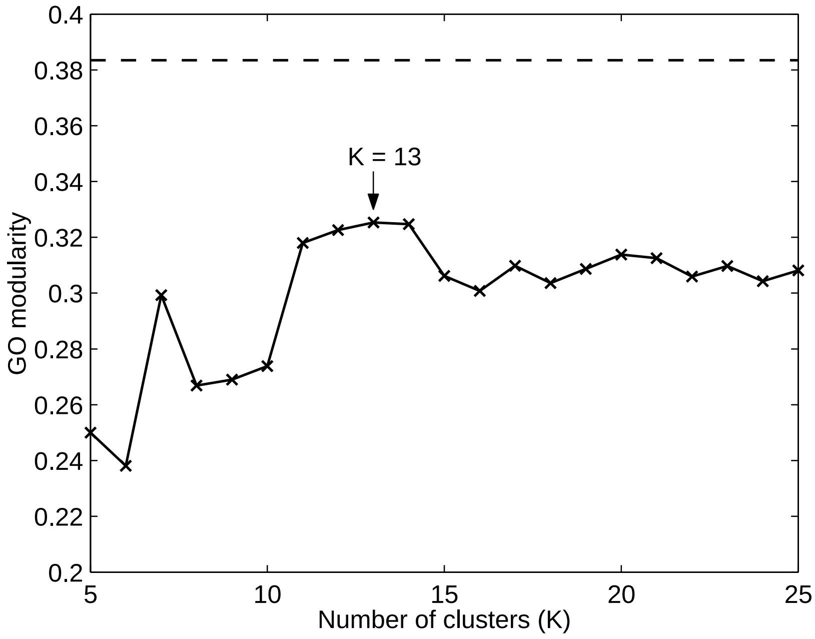

Figure S3: GO-based module score of clusters found by the spectral clustering as a function of the number of clusters (k). The dashed line represents the best score achieved by *Qcut* (with 12 modules).

Table S1: Functional enrichment in gene clusters found by SOTA

Cluster	Size	GO term	Count	Enrichment	P-value
1	679	coenzyme metabolic process	31	3.2	1.80E-09
2	223	nitrogen compound metabolic process	24	3.4	8.70E-08
3	10	-	-	-	-
4	21	-	-	-	-
5	23	meiosis	5	11.6	5.50E-05
6	43	meiosis	7	8.7	1.30E-05
7	14	-	-	-	-
8	13	-	-	-	-
9	22	-	-	-	-
10	499	energy derivation by oxidation of organic compounds	33	3.5	9.20E-11
11	1454	ribonucleoprotein complex biogenesis and assembly	206	2.6	1.20E-49

Table S2: Functional modules in an Arabidopsis co-expression network.

Cluster	Size	GO term	Count	Enrichment	P-value
1	199	-	-	-	-
2	141	-	-	-	-
3	79	-	-	-	-
4	180	catalytic activity	99	1.6	4.1E-09
		amino acid and derivative metabolism	18	4.4	3.9E-08
5	284	endomembrane system	79	1.6	3.5E-06
6	238	oxidoreductase activity	40	2.6	7.7E-09
		secondary metabolism	18	3.1	9.9E-06
7	65	photosynthesis	11	32.6	8.7E-16
8	261	RNA binding	11	4.6	9.2E-06
9	186	galactolipid biosynthesis	3	17.6	1.8E-04
10	19	branched-chain-amino-acid transaminase activity	3	172.6	1.7E-07
11	117	starch metabolism	4	16.0	5.0E-05
		circadian rhythm	6	7.6	8.5E-05
12	271	protein modification	37	2.1	4.3E-06
13	268	methyltransferase activity	8	4.7	1.4E-04
14	13	response to heat	8	87.7	1.9E-15
15	223	antiporter activity	10	6.1	1.5E-06
16	151	transcription regulator activity	60	3.0	2.5E-17
17	200	zeaxanthin epoxidase activity	3	16.4	2.2E-04
18	17	lipid binding	5	48.2	2.9E-08
		membrane	12	2.7	1.8E-04
19	249	calcium ion binding	13	3.2	1.1E-04

Table S3: Modules of cell samples

Module	Sample name	Cell type
1	DLCL-0030, DLCL-0004, DLCL-0029, DLCL-0008, DLCL-0052, DLCL-0034, DLCL-0051, DLCL-0011, DLCL-0032, DLCL-0001, DLCL-0018, DLCL-0037, DLCL-0010, DLCL-0020, DLCL-0003, DLCL-0033, DLCL-0048	DLBCL-1
2	DLCL-0006, DLCL-0049, DLCL-0039, DLCL-0015, DLCL-0026, DLCL-0005, DLCL-0023, DLCL-0027, DLCL-0024, DLCL-0013, DLCL-0002, DLCL-0016, DLCL-0014, Tonsil*, Lymph Node*	DLBCL-2
3	OCI-Ly10, DLCL-0042, DLCL-0007, DLCL-0031, DLCL-0036, DLCL-0025, DLCL-0040, DLCL-0017, DLCL-0028, DLCL-0012, DLCL-0021	DLBCL-3
4	WSU1, Jurkat, U937, OCI-Ly12, OCI-Ly13.2, SUDHL5, OCI-Ly3*, OCI-Ly1*, DLCL-0041*	Transformed cell lines
5	Blood B:memory, Blood B:naive, Blood B, Cord Blood B, CLL-60, CLL-68, CLL-9, CLL-14, CLL-51, CLL-65, CLL-71;Richter's, CLL-71, CLL-13, CLL-39, CLL-52	CLL, Resting blood B
6	FL-9, FL-9;CD19+, FL-12;CD19+, FL-10;CD19+, FL-10, FL-11, FL-11;CD19+, FL-6;CD19+, FL-5;CD19+, SUDHL6*, Tonsil GC B*, Tonsil GC Centroblasts*, DLCL-0009*	FL
7	Blood T:Adult CD4+ Unstim, Blood T:Adult CD4+ I+P Stim, Cord Blood T:Neonatal I+P Stim, Blood T:Neonatal CD4+ Unstim, Thymic T:Fetal CD4+ Unstim, Thymic T:Fetal CD4+ I+P Stim	Blood T cells
8	Blood B:anti-IgM+CD40L low 48h, Blood B:anti-IgM+CD40L high 48h, Blood B:anti-IgM+CD40L 24h, Blood B:anti-IgM 24h, Blood B:anti-IgM+IL-4 24h, Blood B:anti-IgM+CD40L+IL-4 24h, Blood B:anti-IgM+IL-4 6h, Blood B:anti-IgM 6h, Blood B:anti-IgM+CD40L 6h, Blood B:anti-IgM+CD40L+IL-4 6h	Activated blood B cells

*: cells whose types are different from the majority of the cells in the same module
